# Presence of *Helicobacter pylori* and *H. suis* DNA in Free-Range Wild Boars

**DOI:** 10.3390/ani11051269

**Published:** 2021-04-28

**Authors:** Francisco Cortez Nunes, Teresa Letra Mateus, Sílvia Teixeira, Patrícia Barradas, Chloë de Witte, Freddy Haesebrouck, Irina Amorim, Fátima Gärtner

**Affiliations:** 1 Institute of Biomedical Sciences Abel Salazar (ICBAS), University of Porto, 4050-313 Porto, Portugal; franciscojvcnunes@gmail.com (F.C.N.); silvia.goncalves.teixeira@gmail.com (S.T.); fgartnes@ipatimup.pt (F.G.); 2 Institute for Research and Innovation in Health (i3S), University of Porto, 4200-135 Porto, Portugal; 3 Institute of Molecular Pathology and Immunology of the University of Porto (IPATIMUP), 4200-135 Porto, Portugal; 4 CISAS—Centre for Research and Development in Agrifood Systems and Sustainability, Escola Superior Agrária, Instituto Politécnico de Viana do Castelo, 4900-347 Viana do Castelo, Portugal; tlmateus@esa.ipvc.pt; 5 Epidemiology Research Unit (EPIUnit), Institute of Public Health, University of Porto, 4050-091 Porto, Portugal; patricia.barradas@ispup.up.pt; 6 Escola Superior Agrária, Instituto Politécnico de Viana do Castelo, 4900-347 Viana do Castelo, Portugal; 7 Vasco da Gama Universitary School (EUVG), 3020-210 Coimbra, Portugal; 8 Department of Pharmaceutics, Faculty of Pharmaceutical Sciences, Ghent University, 9820 Merelbeke, Belgium; chloe.dewitte@ugent.be; 9 Department of Pathology, Bacteriology and Avian Diseases, Faculty of Veterinary Medicine, Ghent University, 9820 Merelbeke, Belgium; Freddy.Haesebrouck@UGent.be

**Keywords:** one health, wildlife, zoonosis, *Helicobacter* spp., PCR, *Sus scrofa*

## Abstract

**Simple Summary:**

*Helicobacter* *pylori* and *H. suis* are associated with gastric pathologies in humans. To obtain better insights into the potential role of wild boars as reservoirs of these pathogens, gastric samples of 14 animals were tested for the presence of *H. pylori* and *H. suis* DNA. Two wild boars were found PCR-positive for *H. pylori* and one for *H. suis*. This indicates that these microorganisms may colonize the stomach of wild boars.

**Abstract:**

*Helicobacter pylori* (*H. pylori*) is a Gram-negative bacterium that infects half of the human population worldwide, causing gastric disorders, such as chronic gastritis, gastric or duodenal ulcers, and gastric malignancies. *Helicobacter suis (H. suis)* is mainly associated with pigs, but can also colonize the stomach of humans, resulting in gastric pathologies. In pigs, *H. suis* can induce gastritis and seems to play a role in gastric ulcer disease, seriously affecting animal production and welfare. Since close interactions between domestic animals, wildlife, and humans can increase bacterial transmission risk between species, samples of gastric tissue of 14 free range wild boars (*Sus scrofa*) were evaluated for the presence of *H. pylori* and *H. suis* using PCR. Samples from the antral gastric mucosa from two animals were PCR-positive for *H. pylori* and another one for *H. suis*. These findings indicate that these microorganisms were able to colonize the stomach of wild boars and raise awareness for their putative intervention in *Helicobacter* spp. transmission cycle.

## 1. Introduction

The number of infectious diseases has been increasing in humans, with about 60% of those being zoonotic [1]. Of these emerging zoonoses, about 72% are transmitted from wildlife animals [2].

Wild boars are known to be reservoirs of a considerable number of zoonotic bacteria, viruses, and parasites [3], and these infections can be bi-directional (wild/domestic). The exposure to wild boars’ pathogens can happen through different pathways: Direct contact, meat consumption, or indirect intake of contaminated water, food, or through the environment [4]. Changes in human living habits, increased hunting activities, and consumption of wild boar meat play a role in the risk of human exposure to infectious agents [3].

*Helicobacter* species are Gram-negative, spiral-shaped motile bacteria that colonize the gastrointestinal tract of both humans and animals [5,6,7], and have been studied over the years for their association with gastrointestinal diseases [8]. In humans, *Helicobacter pylori* (*H. pylori*) is the most common gastric pathogen, affecting more than half of the world’s population, being responsible for development of gastritis, gastroduodenal ulcers, gastric adenocarcinoma, mucosa-associated lymphoid tissue (MALT) lymphoma, and extra digestive diseases [5,9,10]. In addition, *Helicobacter suis* (*H. suis*) is the most prevalent human gastric non-*Helicobacter pylori Helicobacter* (NHPH) and has been associated with a range of gastric pathologies, including MALT lymphoma, and possibly also extra digestive diseases. Recent reports reinforce that these infections most likely originate from pigs, emphasizing their zoonotic potential [5,11,12,13,14].

In pigs, *H. suis* mainly colonizes the fundic and pyloric gland zone of the stomach [15]. The bacterium presents tropism for the gastric acid-producing parietal cells [16]. Its prevalence appears to be very low prior to weaning, but increases rapidly thereafter, being very high at slaughter age (77%) and in adults (>90%) [15,16,17]. *H. suis* infection causes gastritis, decreased daily weight gain, and plays a role in induction of gastric ulcers, clearly affecting animal production and welfare [18,19,20].

In pigs, there is a report of a natural infection by a *H. pylori-*like bacterium, described as attached to the mucosa of the cardiac and antral portions of the stomach of two out of four healthy young pigs, without gross lesions associated [6,21]. This agent seemed to be morphologically similar to, but antigenically different from *H. pylori*, and its exact identity is not clear [18].

Until now, studies trying to assess the presence of *H. suis* in wild boars (*Sus scrofa*) have been mainly unsuccessful, as described by Flahou et al. [14] and others [22,23].

*Helicobacter* spp. have been described to have zoonotic potential and the close contact between humans, domestic animals, and wild animals deserves more consideration [14,24]. Although reservoirs of wild and domestic animals can be considered as important sources of emerging infectious diseases, it is the human impact on ecological systems that determines the level of risk at the human/animal interface upon the occurrence of emerging zoonotic diseases [14,24].

From an eco-epidemiological perspective, wild boars have an important role in spread of several pathogens [3,4]; thus, the aim of this study was to screen different regions of the stomach (*Pars oesophagea*, fundic, and pyloric gland zone) collected from wild boars for the presence of *H. pylori* and *H. suis*.

## 2. Materials and Methods

### 2.1. Samples Collection

**Figure 1:**
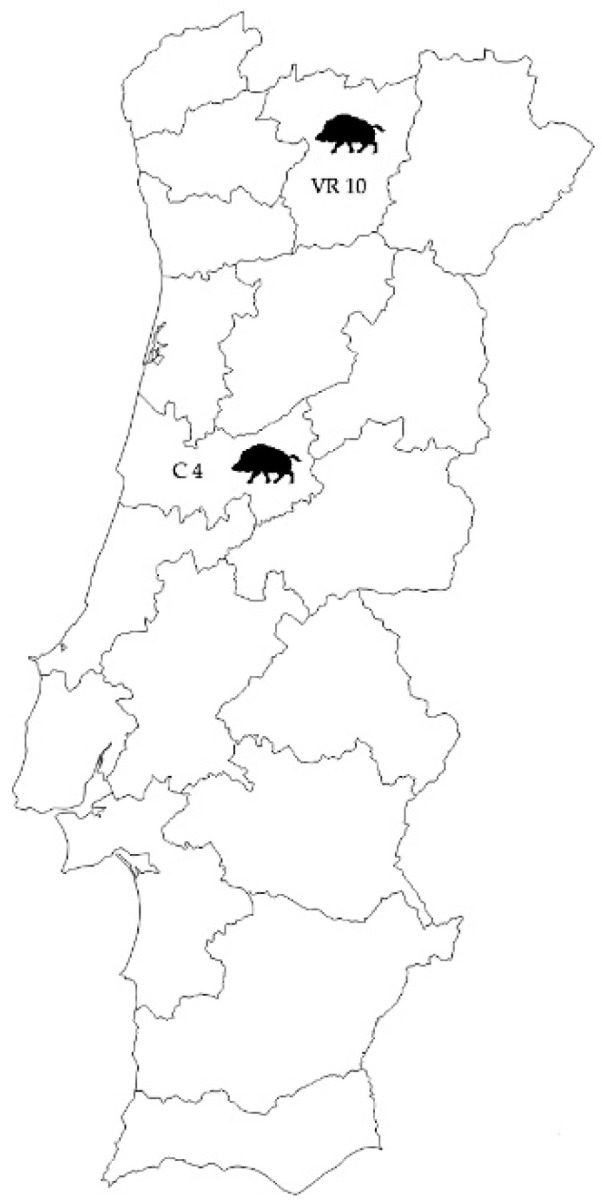
Illustration of the geographical origin of the samples collected in Portugal and evaluated in this study. Samples were collected from 10 wild boars during a national campaign in Vila Real (VR10) and 4 wild boars during a national campaign in Coimbra (C4).

Samples were collected using convenience sampling from fourteen hunted animals during two national campaigns, one in the north and other in the center of Portugal (Vila Real and Coimbra districts, respectively) (Figure 1). All the sampled animals were older than 9 months according to teeth assessment [25,26]. From each animal, gastric samples were collected from three different gastric regions: *Pars oesophagea*, fundic gland zone, and pyloric gland zone (gastric *antrum)* using a Kruuse^®^ Biopsy punch 8 mm. After collection, samples were stored at −20 °C until DNA extraction.

The animals were not slaughtered or euthanized in order to carry out this study, and the fresh gastric tissue specimens were obtained as sub-products derived from the normal activity associated with the meat inspection procedures occurring during these conventional campaigns. None of the actions was performed solely for research purposes and the researchers had no influence on the campaign organization, nor in the meat inspection actions.

### 2.2. DNA Extraction, PCR Conditions and Sequencing

DNA was extracted from 8-mm gastric frozen tissue samples, using EXTRACTME^®^ DNA tissue kit (BLIRT, Poland), according to the instructions provided by the supplier.

All the samples were tested for *H. pylori* and *H. suis* by conventional PCR, according to previously described protocols (Table 1).

Aliquots of each PCR product were electrophoresed on 1.5% agarose gel, stained with Xpert Green Safe DNA gel stain (GRISP, Porto, Portugal) and examined for the presence of a specific fragment under UV light. DNA fragment size was compared with the standard molecular weight, 100bp DNA ladder (GRISP, Porto, Portugal), and the molecular weight of the positive controls (*H. pylori* with 217 and *H. suis* with 150 bp) (Figure S1). As a negative control, distilled water was used. As positive controls, DNA was extracted from pure cultures of *H. pylori* strain 26695 and *H. suis* strain HS1.

To exclude false positive samples, the amplicons from each positive sample were sequenced. Bidirectional sequencing was performed with Sanger method at the genomics core facility of the Institute of Molecular Pathology and Immunology of the University of Porto, Portugal. Sequence editing and multiple alignments were performed with the MegaX Molecular Evolutionary Genetic Analysis version 10.1.8. The sequences obtained were subject to the basic local alignment search tool (BLAST) using the non-redundant nucleotide database (http://blast.ncbi.nlm.nih.gov/Blast.cgi, accessed on 6 January 2021) [28,29].

## 3. Results and Discussion

A total of 42 samples, collected from 14 wild boars, were analyzed.

Based on PCR results, two samples corresponding to two distinct animals were *H. pylori* PCR-positive and another, also corresponding to a different animal, was *H. suis* PCR-positive. These positive samples all originated from the pyloric gland zone (gastric *antrum)* of these animals.

The bidirectional sequencing and BLAST analysis of consensus sequences of partial *ureA* and *ureB* genes showed a homology of 100% with *H. pylori* (GenBank^®^ accession no. AF507994 and AY368264) for two of them and the other positive sample showed a homology of 95.39% with *H. suis* (GenBank^®^ accession no. EF204592).

In the current study, *H. pylori* DNA was detected in the pyloric gland zone of the stomach (gastric *antrum)* from two animals and *H. suis* DNA in the same gastric region of another animal. This region is also a preferential colonization site of *H. suis* in domesticated pigs [17]. These findings might indicate that wild boars are occasionally colonized by *H. pylori* and *H. suis.* It can, however, not be excluded that presence of DNA of these microorganisms might be a consequence of recent contamination; for instance, through contact with domesticated pigs in the case of *H. suis*, or contaminated water or environments for both species. Both *H. pylori* PCR-positive cases did not present any macroscopic gastric alterations. The wild boar stomach where *H. suis* DNA was detected showed signs of mild antral inflammation consisting of mild erythema and congestion at gross examination. Unfortunately, the tissue preservation conditions prevented their detailed microscopic evaluation and the presence of microorganisms with *Helicobacter-*like morphology could not be examined. Further studies using a larger sample and including immunohistochemical and histopathological analysis of gastric tissue from wild boars are absolutely relevant to confirm our findings.

To the authors’ knowledge, this is the first report of *H. pylori* DNA detected in gastric samples of free-ranging wild boars and the first report of *H. pylori* and *H. suis* DNA detection in free-range wild boars in Portugal.

Previously, gastric *antrum* specimens of 17 free-ranging wild boars from Poland were tested, using a *Helicobacter* genus-specific 16S rRNA PCR and sequence analysis of positive samples [23]. In one sample, DNA was detected of a microorganism related to the group of NHPH mainly associated with dogs and cats (previously referred to as *Helicobacter heilmannii* type 2) [23]. More recently, a novel *Helicobacter* species, *H. apri,* was described in wild boars, but this is an enterohepatic *Helicobacter* species [22]. In another survey, very low numbers of *H. suis* were detected in the cardiac gland zone and *Pars oesophagea* from 2 out of 9 wild boars from Belgium. These gastric regions are not the preferential *Helicobacter* colonization sites in the porcine stomach, possibly indicating recent contamination for instance through contact with domesticated pigs or their excretes [14].

Wild boars are known to be reservoirs for several agents for important infectious diseases transmissible to other wild animals, domestic animals, and humans [3,4]. Depending on the pathogen properties and on wild boar density and management, the eco-epidemiological role of these animals can vary from a dead-end over spill over, up to maintenance host [3,4]. Contacts between wild boars and outdoor domestic pigs should be considered a risk for transmission of these pathogens that can directly affect the swine production and animal welfare, since wild boars and domestic pigs belong to the same species (*Sus scrofa*) [3,4,17,19,30].

Hunting dogs and humans can be exposed to wild boars’ pathogens either from fresh carcass contact, handling or consumption of raw, undercooked meat, or indirect contact from contaminated water or environment, and therefore, high risk exposure would include game wardens, hunters, butchers, and other wildlife professional duties [3,4]. Another aspect to consider is the increasing wild boar population and its adaptability to urban areas that can prompt wild boar contact with humans in these areas, making humans more vulnerable to be exposed to wild boars’ pathogens [3,4,31]. The current findings raise *One health* concerns regarding the impact of *H. suis* and *H. pylori* in wild boar welfare, its role and impact on bacterial spread and transmission to the environment, and also to wild and domestic animals, and, ultimately, to humans [3,13,24,31].

## 4. Conclusions

*H. pylori* and *H. suis* DNA was detected in the stomach of free-range wild boars, which might indicate that these animals were colonized by these microorganisms. It can be hypothesized that wild boars might act as reservoirs and contribute to the spread of the *H. pylori* and *H. suis* in the environment, raising a public health concern.

## Figures and Tables

**Table 1:** Primer sequences used for detection of *H. pylori* and *H. suis* and thermo cycling conditions.

Primer	Sequence	Region	Amplicon Size	Cycle			Ref.
				Nr. Cycles	Temp. (°C)	Time	
BFHsuis_F1	5′-AAA ACA MAG GCG ATC GCC CTG TA-3′	*ureA* gene	150bp	40	95 60 72	20 s 30 s 30 s	[5]
BFHsuis_R1	5′-TTT CTT CGC CAG GTT CAA AGC G-3′	*ureA* gene					
BFHpyl_F1	5′-AAA GAG CGT GGT TTT CAT GGC G-3′	*ureAB gene*	217bp	45	94 59 72	30 s 30 s 1 min	[27]
BFHpyl_ R1	5′-GGG TTT TAC CGC CAC CGA ATT TAA-3′	*ureAB gene*					

## Data Availability

The data presented in this study are available on request from the corresponding author

## Supplementary Materials

The following are available online at https://www.mdpi.com/article/10.3390/ani11051269/s1, Figure S1: Example of agarose gel electrophoresis of PCR products of *H. pylori* gene fragments. M, molecular marker; +C, DNA extracted from pure culture of 26695 strain was used as a positive control; WB, wild boar DNA; -C, negative control consisting solely of mix solution (NC).
