# Supplementary material for: Presence of Helicobacter pylori and H. suis DNA in Free-Range Wild Boars
Source: Animals (Basel). 2021 Apr 28;11(5):1269. doi: 10.3390/ani11051269 (PMC8146769; doi:10.3390/ani11051269)
Supplement: Supplementary file 1 [file animals-11-01269-s001.zip › Supplementary data.pdf]

Supplementary data

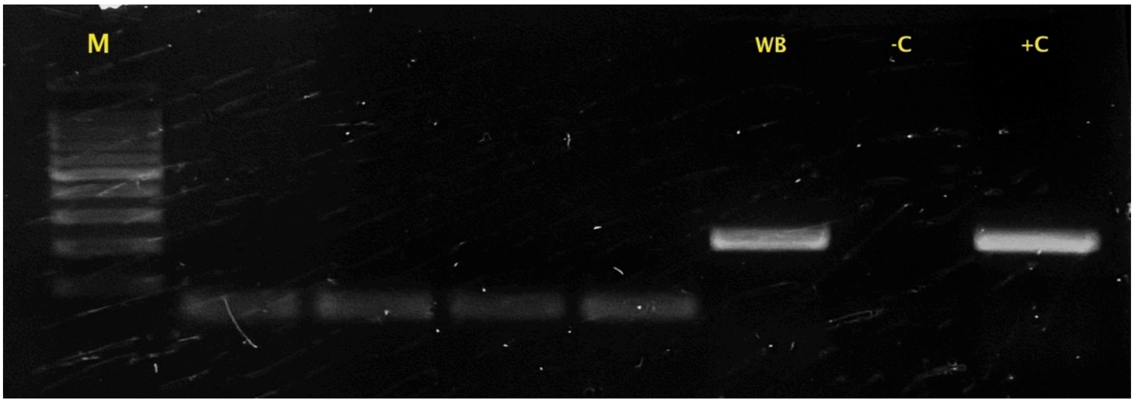

Figure S1. Example of agarose gel electrophoresis of PCR products of *H. pylori* gene fragments**.** M, molecular marker; +C, DNA extracted from pure culture of 26695 strain was used as a positive control; WB, wild boar DNA; -C, negative control consisted solely of mix solution (NC)..
